# Bio-inspired microneedle design for efficient drug/vaccine coating

**DOI:** 10.1007/s10544-019-0456-z

**Published:** 2019-12-16

**Authors:** Cristina Plamadeala, Saransh R. Gosain, Florian Hischen, Boris Buchroithner, Sujitha Puthukodan, Jaroslaw Jacak, Andrea Bocchino, Derek Whelan, Conor O’Mahony, Werner Baumgartner, Johannes Heitz

**Affiliations:** 1 Institute of Applied Physics, Johannes Kepler University Linz, 4040 Linz, Austria; 2 Institute of Biomedical Mechatronics, Johannes Kepler University Linz, 4040 Linz, Austria; 3 University of Applied Sciences Upper Austria, Campus Linz, 4020 Linz, Austria; 4 Tyndall National Institute, University College Cork, T12 R5CP Cork, Ireland

**Keywords:** Two-photon polymerization, Directed fluid transport, Microfluidics, Microneedles, Biomimetics

## Abstract

Biomimetics is the interdisciplinary scientific field focused on the study and imitation of biological systems, with the aim of solving complex technological problems. In this paper, we present a new bio-inspired design for microneedles (MNs) and MN arrays, intended for rapidly coating the MNs with drug/vaccine. The biomimetic approach consists in ornamenting the lateral sides of pyramidal MNs with structures inspired by the external scent efferent systems of some European true bugs, which facilitate a directional liquid transport. To realize these MNs, two-photon polymerization (TPP) technique was used. Liquid coating capabilities of structured and non-structured MNs were compared. Moreover, both in-vivo and ex-vivo skin tests were performed to prove that MNs pierce the skin. We show that the arrays of MNs can be accurately replicated using a micro-moulding technique. We believe this design will be beneficial for the process of drug/vaccine loading onto the needles’ surfaces, by making it more efficient and by reducing the drug/vaccine wastage during MN coating process.

**Electronic supplementary material:**

The online version of this article (10.1007/s10544-019-0456-z) contains supplementary material, which is available to authorized users.

## Introduction

Microneedles (MNs) are sharp, needle-like projections, generally less than 1 mm tall and arranged in arrays covering surfaces ranging from a few square millimetres up to several square centimetres (Larrañeta et al. 2016). Due to their short dimensions and correspondingly shallow penetration depths (10–80% of their length (Römgens et al. 2014)), MNs are able to penetrate into the outermost epidermal layers, without irritating the nerve endings or reaching the blood vessels that lie deeper in the skin. Consequently, MNs are minimally invasive, and do not trigger pain sensations nor induce bleeding (Jeong et al. 2017). Therefore, it is expected that MNs will find wide-ranging applications in transdermal drug and vaccine delivery (Larrañeta et al. 2016).

Generally, based on their overall geometry and mechanism of drug delivery, microneedles can be divided into four different types, i. e. solid, coated, dissolving, and hollow MNs (Kim et al. 2012). Solid MNs are used for skin pre-treatment, i. e. for creating micro-pores in the skin. Subsequently, a topical formulation is placed onto the pre-treated area and it diffuses through the micro-pores into the skin (Nguyen et al. 2016). Coated MNs are covered with a thin layer of formulation before applying them onto the skin. Upon penetrating the skin, the formulation dissolves and releases the active ingredients (Gill and Prausnitz 2007). Dissolving MNs are made up of polymeric materials mixed with an active agent. When inserted into the skin, they rapidly degrade, releasing the drug or vaccine cargo (Leone et al. 2017). Hollow MNs, similar to hypodermic injections, have an opening through which the liquid formulation is injected into the skin (Ita 2018).

One of the most common methods used for coating microneedles with a drug formulation is the dip coating technique: the MN patch is immersed in a solution, then withdrawn, and dried. Even though the procedure is simple, it has several disadvantages: the immersion may last a long time; the thickness of the coating layer is not uniform (making it complicated to accurately dose the drug); and finally - due to gravitational force - the liquid drug formulation tends to accumulate at the base of the MNs, resulting in drug loss (Haj-Ahmad et al. 2015). These disadvantages determined the development of new coating techniques, such as gas-jet drying (Chen et al. 2009), spray drying (McGrath et al. 2011), electrohydrodynamic atomisation (Khan et al. 2014), and ink-jet printing (Boehm et al. 2014). While these techniques allow a more precise and uniform drug deposition, with reduced drug wastage, they require rather sophisticated equipment and trained personnel.

Biomimetic thinking has brought considerable improvement to the field of microneedle design. Substantial work is being carried out towards understanding the mechanism of female mosquitos’ proboscis (mouthparts) insertion into skin. This knowledge was implemented in MN design and production (Kong and Wu 2010). It has been shown by different research groups that mosquito bio-inspired MNs need considerably lower insertion forces to penetrate the skin (Aoyagi et al. 2008). Similar results were obtained with MNs mimicking the North American porcupine quills (Cho et al. 2012). Apart from that, MNs with swellable tips inspired by the proboscis of an endoparasite (*Pomphorhynchus laevis*) were proven to be efficient in the adhesion to, and interlocking with the soft tissues. These MN patches are much more flexible than conventional polymer-based MNs, therefore they are easily removed from the tissues, without damaging the tissue or the MNs (Yang et al. 2013).

Biomimetic approaches could also be of significant use in MN drug/vaccine coating. Nature inspired surfaces with special wetting properties range from ultra-hydrophobic self-cleaning applications of the lotus-effect (Barthlott and Neinhuis 1997) up to hydrophilic polymer anti-fouling coating applications inspired by marine mussels (Hamming and Messersmith 2008). In the context of MN coating, bio-inspired microfluidic devices and structured surfaces which, coming in contact with different liquids, promote a directional movement of the liquids (Comanns et al. 2014; Buchberger et al. 2015; Comanns et al. 2015; Comanns et al. 2016; Kirner et al. 2017; Plamadeala et al. 2017; Buchberger et al. 2018; Hischen et al. 2018), are of great interest.

In our previous work, we have developed artificial surface structures inspired by the external scent efferent system of some European true bugs (Hischen et al. 2018). These bugs use oriented microstructures for unidirectional transport of defensive liquids on their body surfaces (from the places where the liquids are secreted to the places where they are evaporated). We have previously shown that technical surfaces (such as polymers or steels) with open capillary channels containing scaled up bug-inspired oriented structures are able to transport liquids (such as soapy solutions or oils) in the direction the tips of the structures point to, while halting the transport in any other direction. We also reported that similar self-organised conical structures inspired by the external scent efferent system of European true bugs obtained on polyimide foil (with dimensions in the range of tens of micrometres) were able to sustain the directional liquid movement of soap-water solution even when tilted (Hischen et al. 2018). Similar microstructures, when placed in a closed capillary channel configuration, guide the movement of oily liquids in the direction against the tips of the structures (Plamadeala et al. 2017).

In this paper, we have combined rapid prototyping techniques and microneedle fabrication technologies towards the specific goal of a MN design capable of being quickly and easily coated with a uniform layer of drugs or vaccines. We accomplished this by ornamenting the lateral faces of pyramidal MNs with bio-inspired microstructures, which facilitate the directional transport of the liquids from the bottom of the MNs towards their tips.

To create the microneedles, two-photon polymerization (TPP) lithography was used – an intrinsically three-dimensional (3D) micro-fabrication technique, which enables production of micro-scale devices with feature sizes below the resolution limit (Tanaka et al. 2002). This method is largely implemented to create MNs and MN arrays, as it offers the advantage of generating structures of user-defined designs, sizes, and distributions (Gittard et al. 2010). Wetting tests have shown that the bio-inspired structures transport fluid towards the tip of the needle, as intended. A micro-replication technique (O’Mahony et al. 2016) was used to quickly produce copies that are geometrically indistinguishable from the master templates. The replicas were also used to demonstrate skin penetration through ex-vivo skin studies. To our knowledge, this work represents the first MN design which makes use of bio-inspired surface structures for passive liquid transportation on its lateral surfaces. This paper is a preliminary engineering study that focusses on the manufacturing challenge of writing microstructures on very steep sidewalls, which lead to a fluid transport to the needle tip, and then carries out preliminary work to assess feasibility.

## Materials and methods

### Computer aided designs

A complete test sample consists of three parts – a guiding channel, and two different MNs. All the computer-aided designs (CADs) were realized using SketchUp Make 2017 – a freeware for three-dimensional (3D) modelling.

**Fig. 1:**
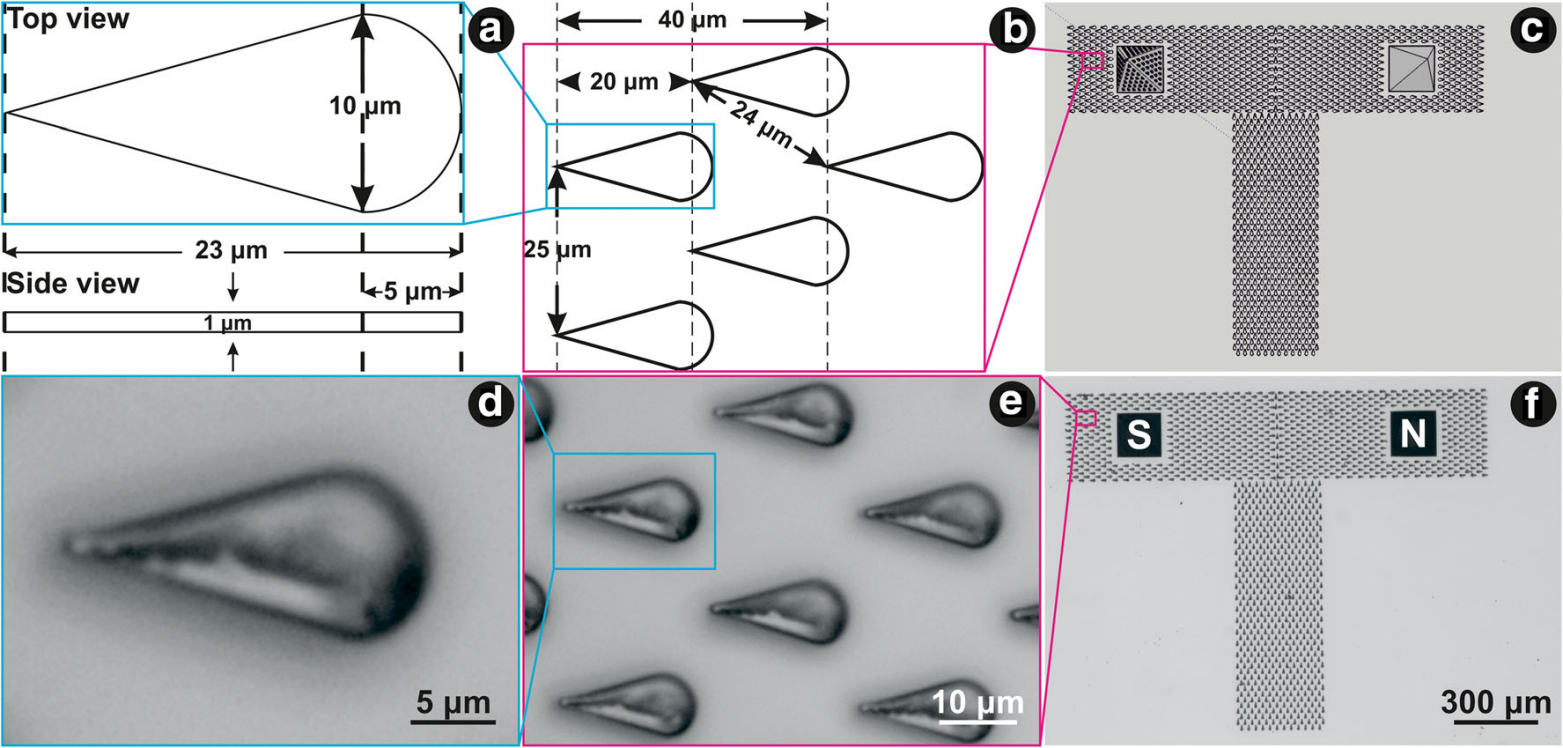
Computer-aided designs of (**a**) a single microstructure (top and side view); (**b**) microstructures and the distances between them in different directions; (**c**) the whole test sample - T channel and two MNs – structured (S) and non-structured (N). Optical microscope images of (**d**) an individual microstructure; (**e**) several microstructures, and (**f**) the whole test sample

The channel, consisting of periodic bug-inspired microstructures (Fig. 1a, b), is intended to guide the fluid to the base of the MNs in a controlled manner (Fig. 1c). Each bug-inspired microstructure had a length of 23 μm, a base width of 10 μm and a height of 1 μm (Fig. 1a). Length to base width ratio was kept 2.3 to 1, as described in (Hischen et al. 2018). Distance between the tips of the two consecutive microstructures within one row was set to 25 μm, and the distance between the tips of two microstructures from two consecutive rows was set to 24 μm (Fig. 1b). The whole design of the channel resembles the letter T. Each of the two ‘arms’ had a square gap with an area of about 170 × 170 μm^2^, into which the MNs were fabricated. The microstructures building up the ‘arms’ of the vertical channel are oriented towards the MNs. The vertical channel measures 900 μm in length, the horizontal channel measures 1500 μm in length, and both channels have a width of 300 μm.

**Fig. 2:**
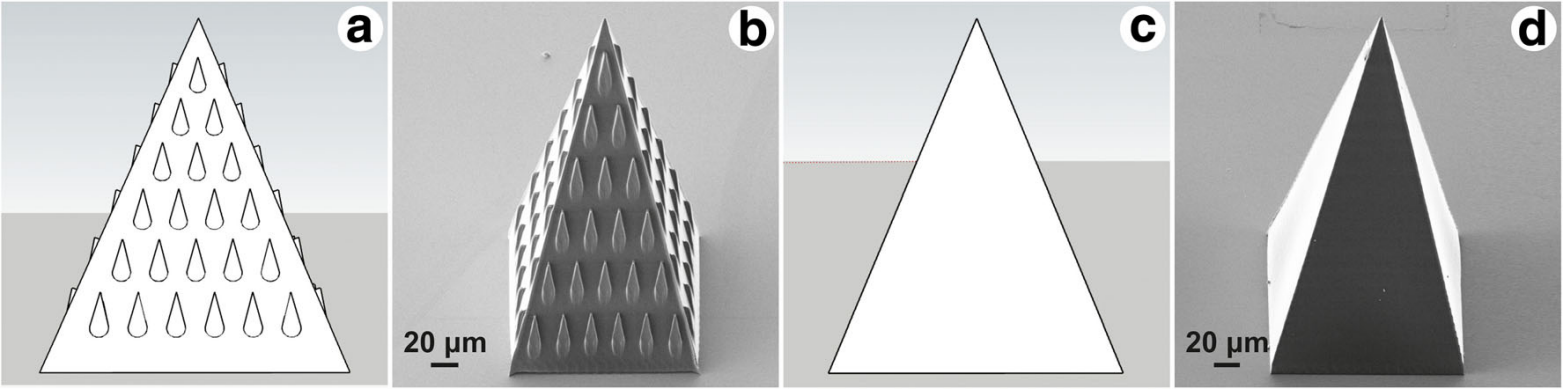
Computer-aided design of (**a**) the structured MN, and (**b**) the non-structured MN. The corresponding SEM images of (**c**) the structured MN, and (**d**) the non-structured MN

The MNs were designed as 210 μm high pyramids with square bases, 160 μm long sides and 10 μm thick walls (Fig. 2a, c). The structured MN had the lateral triangular faces ornamented with bug-inspired microstructures. Each microstructure was 20 μm long (slightly shorter than the microstructures in the guiding channel, due to the expected elongation on z-direction), with a base width of 10 μm and a tip height of 5 μm, keeping the same length: width: height ratio as in (Hischen et al. 2018). The distance between two consecutive rows was set to 30 μm.

### Two-photon lithography setups

To write the guiding channel, a custom-made setup (Ti-sapphire femtosecond laser (Mai Tai, Spectra Physics), wavelength = 800 nm, pulse length = 150 fs, repetition rate = 80 MHz) was used. The setup is described in more detail in (Plamadeala et al. 2017). The channels were written on a #1.5 glass substrate, with an area of 24 × 50 mm^2^, and thickness of 170 ± 30 μm (Menzel-Gläser coverslips) at a speed of 20 μm s^−1^, with a laser excitation power of 12 mW.

To write the MNs, a commercially available setup, provided by the Workshop of Photonics® was used. The 515 nm laser beam, with a repetition rate of 1 MHz, and 290 fs pulse duration, was focused into the photoresist by an objective lens with 50-fold magnification and 0.42 numerical aperture. The MNs were written layer by layer (layer thickness = 1 μm) in a sandwich sample configuration (Serbin et al. 2004). For the non-structured MNs, a writing speed of 3 mm s^−1^ and a power of 2.4 mW were used; for the structured MNs, a speed of 1 mm s^−1^ and a power of 1 mW were used, both measured in front of the objective lens.

### Photoresists

The photoresist used for the microstructured guiding channels was OrmoComp® (micro resist technology GmbH). The photoresist used for MNs consisted of OrmoComp® and 1 wt% of Ciba® IRGACURE® 2959 (Sigma-Aldrich). In both cases, the unpolymerized photoresist was dissolved in OrmoDev developer (micro resist technology GmbH).

### Wetting tests and fluorescence microscopy imaging

Wetting tests were performed to prove the wettability of the lateral sides of the structured MNs. The liquid used for wetting tests and fluorescence microscopy imaging was a mixture of 1 to 10 of an ethanol solution of Alexa Fluor® 555 fluorophore (Invitrogen™, ThermoFisher Scientific Inc.) and distilled water. Furthermore, less than 1% of soap (DAWN liquid dish soap) was added, in order to adjust the liquid contact angle in a range of 40° to 50°, which is a prerequisite for the directional liquid movement (Hischen et al. 2018).

**Fig. 3:**
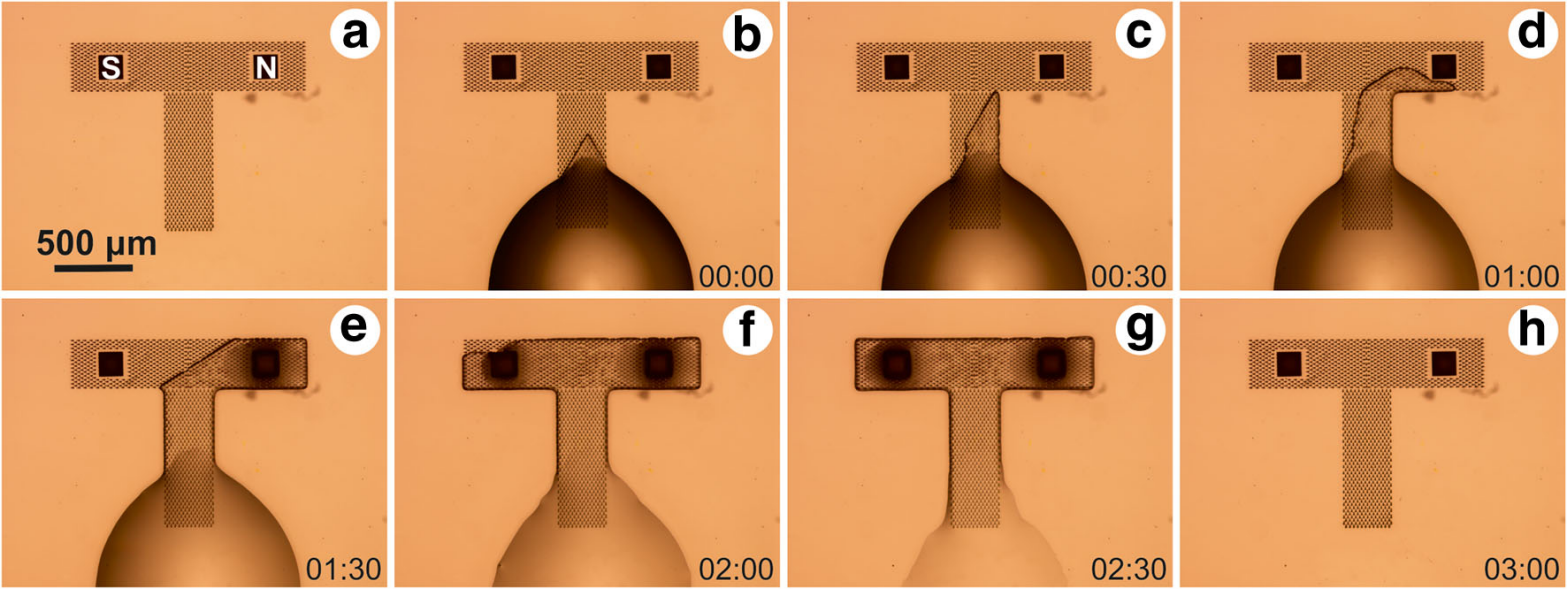
**a** Microscope image of the sample before the wetting test. **b**–**h** Liquid front movement in the guiding channel, captured every 30 s following liquid deposition at the bottom of the guiding channel (**b**) until complete evaporation of the liquid (H)

For fluorescence analysis, a Nikon Eclipse T*i*-U microscope (Nikon Instruments Inc.), with a Texas Red filter cube, with excitation window at 540–580 nm, and emission window at 600–660 nm (Nikon Instruments Inc.) was used. Before performing the wetting test, the samples were imaged in the red channel, to assess their auto-fluorescence. Afterwards, a small volume of water-fluorophore solution was deposited in the close proximity of the microstructured channel (Fig. 3b). After the wetting test, that is, after the complete evaporation of the liquid, the samples were again imaged in the red channel. Fluorescence microscope images were recorded and line intensity profiles were obtained using the ImageJ software.

### Replication

For mass-production of MNs, a micro-replication process, which is fast and cost-efficient, is necessary. To prove that even the features of the structured MNs can be replicated with high accuracy and no damage, arrays of 3 × 3 MNs (spaced 750 μm apart) printed on a glass slide were replicated as described in (O’Mahony et al. 2016). The master arrays were gold sputter coated prior to replication, and imaged with a scanning electron microscope (SEM). The gold layer is also helpful for the last step of replication, as it assists the demoulding.

The glass slide containing the MNs was attached to a polystyrene Petri dish using Super Glue (Loctite Superglue). After the glue had dried, a mixture of 10:1 of the two components of polydimethylsiloxane SYLGARD 184 (Neyco) was poured inside the Petri dish, covering the MNs array. The Petri dish was then left overnight on a levelled shelf, and cured in the morning at 80 °C for 1 h.

In a similar way, the two components of EPO-TEK® 353ND (Epoxy Technology, Inc.) were mixed together in a ratio of 10:1, degassed, and used to fill the SYLGARD® 184 mould. A flat disk of PDMS was used as rear mould and the ensemble was put on top of a vacuum table covered by a 500 g weight for 1 h. The EPO-TEK® 353ND was cured at 80 °C for 1 h and left at room temperature for 14 h to cool down. Finally, the two moulds peeled away from the MNs patch.

The master template (the original MNs array), as well as the epoxy replicas, were SEM imaged to assess the fidelity of the replication process. From the SEM images, the MNs’ base lengths, tip widths, as well as the heights, widths and lengths of individual microstructures were measured using the ImageJ software.

### Skin penetration

To confirm skin penetration, MNs were used for two sets of skin tests. Following protocols approved by the Clinical Research Ethics Committee of the Cork Teaching Hospitals (CREC), human skin was excised following plastic surgical operations and stored at −80 °C until needed. The skin was thawed and incubated in phosphate-buffered saline (PBS) solution (Sigma-Aldrich) for 30 min at 32 °C. Then, the skin samples were trimmed of the excess fat, cut into squares of approximately 3 cm × 3 cm and mounted on am artificial tissue substrate (Wound Closure Pad, Limbs & Things, Bristol, UK), to mimic the mechanical properties of underlying tissues. A custom-built, spring-loaded applicator, with a spring constant of 274 N/m, and impact energy of 0.4 ± 0.01 J/cm^2^ was used to apply the epoxy MNs array with a surface of 4 × 4 mm^2^ to the skin in a repeatable and reliable manner. After the impact, the applicator maintained a force of 0.5 N on the MNs array, and retracted after 2 min. The test was repeated three times to confirm the reproducibility of the results. After each test, the impact areas were stained with methylene blue (Sigma-Aldrich) for 10 min, prior to PBS rinsing and tape stripping to remove the excess dye present on the surface of the skin (Sun et al. 2002). Optical microscope images of the impact areas and of the MNs after insertion were recorded.

For a second set of skin tests, an optical coherence tomography (OCT) system was used to observe the penetration depth of the MNs. The MNs array was pressed against the forearm skin of a healthy male volunteer and fixed with tape prior to recording the images. The commercially available TELESTO™ Spectral Domain OCT imaging system (Thorlabs, Inc.) using a super-luminescent diode, with a central wavelength at 1325 nm, with a 150 nm bandwidth was employed. The device offers a lateral resolution of 15 μm and an axial resolution of less than 7.5 μm. After performing several skin tests, the MNs were imaged using a scanning electron microscope.

## Results and discussion

Figures 1a–c show in detail the computer-aided design of the bug-inspired microstructures which make up the guiding channel. Figure 1c shows the guiding channel and two different MNs – the structured MN on the left-hand side and the unstructured MN on the right-hand side (used as negative control sample). Figures 1d–f show the corresponding optical microscope images of the bug-inspired microstructures and the whole T-array, after the developing process. One microstructure of the guiding channel has a length of 21.20 ± 0.47 μm, base width of 9.95 ± 0.46 μm, and a tip width of 1.21 ± 0.22 μm. The difference from the designed length is due to a post-curing shrinkage of up to 7% (OrmoComp® data sheet). Due to the ellipsoidal voxel shape in TPP writing (Sun et al. 2002; DeVoe et al. 2003; Fischer and Wegener 2011), an elongation of the microstructures in the z-direction occurred. Therefore, the average height of the microstructures was 14.65 ± 2.26 μm.

The CAD designs of the structured and non-structured MNs are shown in Fig. 2a, c, respectively. The corresponding SEM micrographs are presented in Fig. 2b, d, respectively. Both MNs have tips of about 1 μm in diameter, and base lengths of 150 μm (post-curing shrinkage). The bug inspired microstructures on the lateral surfaces of the structured MN (Fig. 2c) are regular and well defined. Post-curing shrinkage and the voxel elongation in z-direction influenced the dimensions of the micro-ornamentation on the MNs lateral sides. Measurements revealed average tip height of 3.94 ± 0.21 μm, tip width of 2.01 ± 0.24 μm, base width of 9.81 ± 0.22 μm, and length of about 31.42 ± 0.61 μm.

**Fig. 4:**
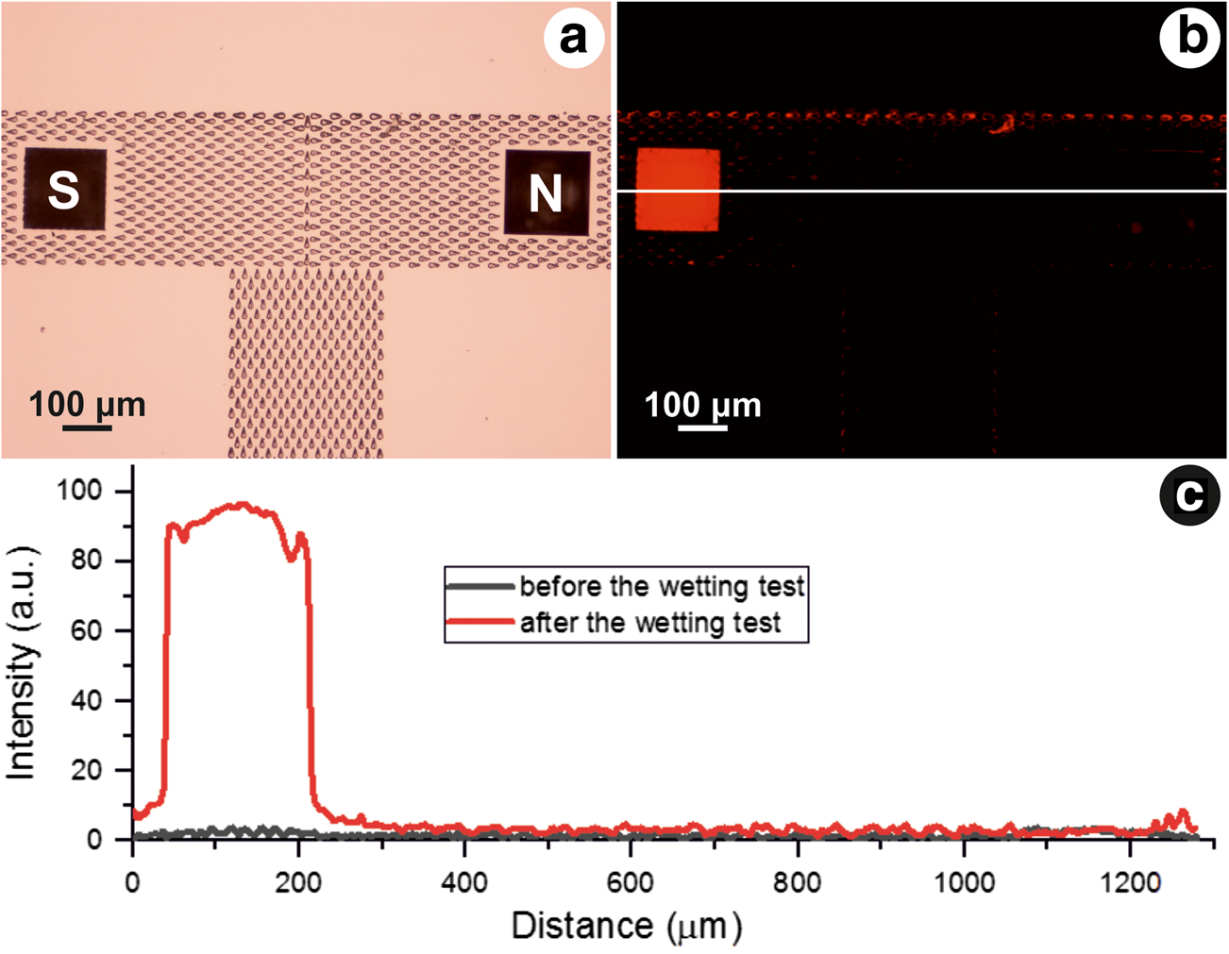
**a** Optical microscope image of the T-shaped channel and the two microneedles after the wetting test. **b** Fluorescent microscope image taken in the red channel after the wetting test performed with a fluorophore-soap-water solution. **c** Intensity profile along the yellow line in Fig. 4b (before and after the wetting test)

An image sequence of the liquid movement in the guiding channel is presented in Fig. 3. The droplet was deposited at the lower rim of the vertical channel (Fig. 3b), and within the following two minutes the liquid front reached the MNs (Fig. 3f, g). As seen in Fig. 3d, e, the liquid front had a tendency of moving towards the right-hand side of the T-array. Therefore, the liquid front does not come in contact with both MNs in the same time. This was a systematic error, encountered in every experiment. It was a result of a slightly tilted glass slide during the writing process, causing the microstructures height difference within the array. To counteract this effect, for each sample with a structured MN on the left-hand side and the not-structured MN on the right-hand side, a sample with switched position of the two MNs was tested. In all cases, the fluorescent microscope analysis was consistent with the results shown in Fig. 4c.

Figure 4b, c present in summary the results of the wetting tests performed with a soap-liquid solution containing a fluorescent dye. Figure 4b shows an intense fluorescent signal originating from the structured MN (left-hand side), in contrast to the non-structured MN (right-hand side), whose edges are barely observable. Confocal microscope imaging of the non-structured MN after the wetting test (Supplement 1) showed a 30–40 μm high liquid front at the MN’s base. This corresponds to the 15 μm high liquid front moving in the structured guiding channel, and additional capillary action manifested by the liquid front when it encounters the MN’s edges. On the other hand, confocal microscope imaging of the structured MN after the wetting test (Supplement 2) showed the fluorescence of the MN on the whole z-axis travel range of 100 μm. These results are consistent with the ones presented in Fig. 4b. It is clear that the surface structures on the structured MN aid the liquid front movement on its lateral sides, justified by the intense fluorescence signal on the structured MN, compared to the non-structured one.

Figure 4c shows the intensity profile along the white line shown in Fig. 4b, before the wetting test (grey curve) and after the wetting test (red curve). Low auto-fluorescence before the wetting test is observed. The fluorescence detected from the non-structured MN after the wetting test showed intensities in the same range as the structures of the guiding channel (due to comparable height of the liquid front). In contrast, the intensity detected from the structured MN rose up to 100. The wetting tests were repeated several times. The results were consistent – no or very little fluorescence recorded from the non-structured MN, and high signals recorded from the structured MNs.

MNs with the same bug-inspired ornamentation, but pointing downwards, were also tested (Supplement 3). According to (Hischen et al. 2018), the stopping liquid front needs to create a V-shaped front before halting completely. In the case of MNs, the V-shaped liquid front would be formed on the MN itself, and hence the fluorescence signal after the wetting test is comparable to the MN with surface structures pointing upwards. Also according to (Hischen et al. 2018), the velocity of the stopping liquid front (against the tips of the microstructures) is considerably slower than that of the advancing liquid front in the direction of the tips of the microstructures. Therefore, this study focuses only on the MNs with upwards pointing microstructures.

**Fig. 5:**
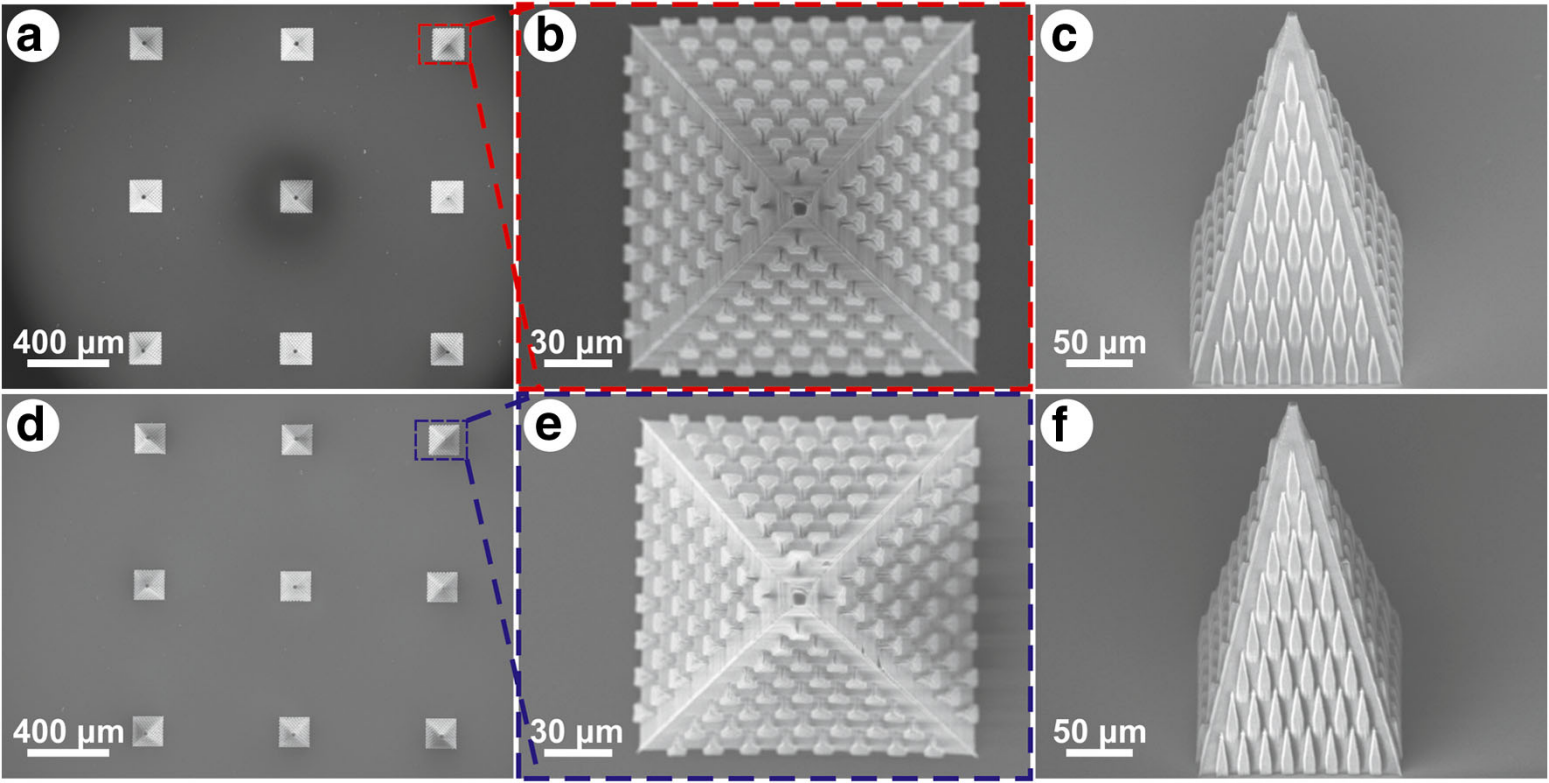
Scanning electron micrographs of the 3 × 3 MNs array of (**a**) the TPP original, and (**d**) its corresponding epoxy replica. SEM micrograph of a TPP structured MN from the 3 × 3 array: (**b**) top view, and (**c**) side view. SEM micrograph of an epoxy replicated structured MN: (**e**) top view, and (**f**) side view

Figure 5 shows scanning electron micrographs of the 3 × 3 array of MNs produced with TPP, imaged after replication (Fig. 5a) and its corresponding replica in EPO-TEK® 353ND (Fig. 5d). After the replication process, all the MNs on the master template were intact on the glass substrate. A more detailed top and side views of the original MN can be seen in Fig. 5b, c, and the equivalent replicated MN in Fig. 5e, f, respectively. The MNs in this array had slightly different lengths and distributions of the micro-ornamentations than earlier presented in Fig. 2c. Despite being densely packed, the surface structures on the replicated MNs are very well defined

Measurements of the MNs’ base length yielded an average value of 155.01 ± 1.69 μm for the original one, and a value of 150.51 ± 1.38 μm for the replicated MN. The difference is due to post curing shrinkage. The tip diameters for both of MNs were about 6 μm. Microstructures’ lengths, base and tip widths mean values and standard deviations (*n* = 36) are summarised in Table 1. Student *t*-test revealed no significant difference between the three sets of data, as the *p*-values were always greater than 0.1. These results show a successful and exact replication of the MNs array.

**Table 1:** Mean values and standard deviations of the bug-inspired microstructures lengths, base and tip widths. The parameters were measured on the 36 microstructures, visible on the lateral faces of the original MN (Fig. 5c) and its corresponding replicated MN (Fig. 5f)

	Length (μm)		Base width (μm)		Tip width (μm)	
	Original	Replica	Original	Replica	Original	Replica
Mean value	39.66	39.7	9.72	9.69	2.15	2.24
Std. deviation	0.71	0.89	0.37	0.35	0.33	0.31

**Fig. 6:**
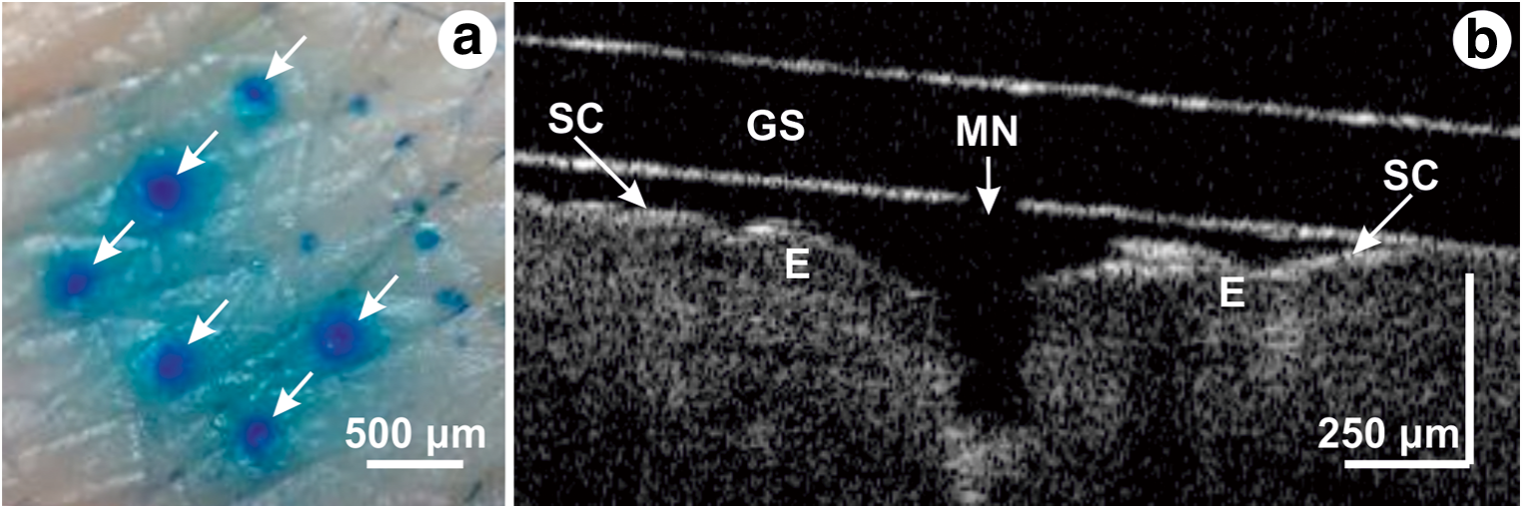
Skin penetration tests. **a** Methylene blue stained marks after the skin test. **b** Optical coherence tomography of one MN (SC – stratum corneum, MN – microneedle, GS – glass substrate, E – epidermis)

Figure 6 shows in summary the results of the skin tests performed on ex-vivo human skin and on a male volunteer. Each of the skin tests performed with the epoxy MNs array showed five to six penetration marks. Figure 6a shows six methylene blue stained marks. The number of penetration marks and their dimension can be explained by the irregularity and elasticity of the skin surface. Another possible explanation might be the poor penetration of some the needles.

Optical coherence tomography analysis of the skin (Fig. 6b) allowed to discriminate between distinct skin layers and even sublayers (Sattler et al. 2013). The outermost sublayer, the stratum corneum (SC), appears as a thin intense line due to the presence of keratin. The epidermis (E) is located right below the SC, and gives a less intense OCT signal. OCT analysis showed that when being pressed against the skin, the MNs penetrated to a depth of about two thirds of their height, corresponding to similar results presented in (Enfield et al. 2010). Nonetheless, MNs had sufficient impact energy and sharpness to break the SC barrier and penetrate through to the epidermal layer. OCT images were also recorded after the MN patch was removed (not shown). The penetration marks were much smaller than the MN dimensions, in agreement with the results from Fig. 6a, most likely due to skin’s elastic properties. No pain sensations were reported by the volunteer.

Optical microscope and scanning electron microscope analysis of the MNs (data not shown), which were subjected to several skin tests, showed no damage caused by the tests. This confirms the mechanical stability of the MNs: the MNs did not break and presented no risk of remaining in the skin.

The wetting tests were performed using a water-based liquid on OrmoComp® MNs. To obtain a directional flow, it is essential that the dimensional parameters (structures heights and curvatures) are set in the correct range, and that the liquid contact angle is adjusted under 45°. As discussed in detail in (Hischen et al. 2018), the microstructures parameters and the contact angle define the shape of the fluid front around each microstructure. When the liquid fronts of neighbouring microstructures touch, a fluid transport in the forward direction occurs. The transport velocity is mainly determined by liquid viscosity, as these velocities allow considering a laminar flow conditions.

A general problem when applying drugs via a MN patch is to correctly dose the administered drug, as the needles loading is a delicate process (Haj-Ahmad et al. 2015). Several approaches are discussed to find better solutions (Chen et al. 2009; McGrath et al. 2011; Khan et al. 2014; Boehm et al. 2014). The use of microstructures might reveal a new strategy. As seen in Supplement 2, the fluorescent agent is mainly concentrated at the microstructures rims, at the bottom of the channel and on the MN’s lateral faces. This effect is due to solvent evaporation in the capillaries surrounding each microstructure, leaving only the non-volatile ingredients. The liquid volume in each capillary channel around identical microstructures on the MN lateral faces should be approximately the same (at least under idealized conditions (Hischen et al. 2018)), depending on the contact angle and the structure geometry. Thus, the liquid loaded on a MN with a certain number of microstructures can be defined as the total volume of fluid that can be hold within the microstructures capillary network, minus the volatile part of the solvent. This obviously requires a known concentration of the pharmaceutical agent in the solvent and the constriction that the agent is non-volatile. How much of the active agent is in the end transferred into the tissue of the patient during application is an open question, which is the case for several other loading techniques discussed in literature.

Immersion coating (i.e. the entire array is immersed in the formulation) is the simplest of all coating techniques, but it leads to significant wastage of the active ingredient, since coatings are produced on the entire microneedle patch and only a small percentage is actually delivered (Matriano et al. n.d.). Instead, dip coating is a preferable method of coating microneedles that involves the partial immersion of the tip region of the needle into the coating formulation, so that the base is not needlessly coated. However, it generally requires the construction of precise equipment capable of accurately and repeatedly lowering an array into liquid with microscopic precision, and this process may not be scalable to a high-volume manufacturing environment. High-viscosity solutions are typically used during the coating process, as this is an effective method of increasing drug loading, but it may also result in the formation of a large bulb of dried formulation at or near the tip, which leads to reduced tip sharpness and makes skin penetration difficult (Chen et al. 2017; Kim et al. 2010). In response to those limitations, this work proposes the use of novel microstructures that could lead to uniform coating on the lateral faces of the microneedle, without significantly altering the geometry or tip sharpness of the microneedle and without waste of active ingredient.

As mentioned above, the performance of the structures is dependent on contact angle and the viscosity of the formulation must be adjusted to keep the contact angle under 45°, with sufficient transport velocity. While this early engineering proof-of-concept work has not considered specific drugs or vaccines, we expect that coating of a wide range of formulations should be possible once the contact angle constraints are met. A good review of the active materials that have been coated onto microneedles to date is available in (Ingrole and Gill 2019).

We do not think that the structures themselves will be critical for drug release and delivery, but will rather aid in uniform loading of the drug cargo onto the needle surfaces. It is preferable that drug loading is concentrated around the lateral surfaces of the microneedle, and around the upper portions in particular (see Fig. 2 of (Ingrole and Gill 2019)), as a number of works have shown that microneedles do not penetrate to their full height due to skin elasticity and compression, and that penetration depth is generally limited to between half and two-thirds of needle height (Enfield et al. 2010; Ripolin et al. 2017). Because of this, it is desirable that drug cargo is not located around the lower regions of the needle, as it does not enter the skin and is therefore wasted. Similarly, drug coatings on the base of the needle array (and in this case, in the lateral flow channels) will also be wasted. Because of this, future work will investigate the use of carefully controlled volumes that will travel from the channel to the upper half of the needle with the aim of increasing the percentage of delivered cargo and reducing the level of wasted formulation. We do not envisage the structures themselves affecting drug delivery, as the skins should conform to the relatively low heights of the microstructures. Furthermore, interstitial fluid could even collect around any gaps that may remain, and may even aid dissolution of the coating.

## Conclusion

As microneedles are becoming a more auspicious tool for transdermal drug delivery, a large amount of research is being carried out to optimize MN aspect ratios, fabrication materials and techniques, as well as the methods used for coating their surfaces with drug and vaccine formulations. In this study we describe a novel biomimetic approach towards drug delivery and show proof of principle. We introduced a new MN design - the lateral faces of the MNs were ornamented with bug-inspired microstructures. When compared to the non-structured negative control MN, the structured MN proved to be easily loaded with liquid. We showed that the MN arrays can be rapidly replicated, with high accuracy, in medical-grade epoxy materials. Skin tests proved that the MNs are able to break the stratum corneum barrier and reach the epidermis, with no damage to the MNs, and no pain sensation for the volunteer. However further research is required to better characterise and demonstrate pharmacological loading capabilities of the MN and drug delivery efficiency both in vitro and in vivo models.

In the future, we envisage an array of hundreds of MNs, interconnected by a net of guiding channels, consisting of bug-inspired structures. The arrays would be easily loaded with necessary drugs/vaccines, without recourse to complicated deposition procedures, and with minimal formulation wastage. To our knowledge, this is the first MN design that makes use of bio-inspired surface structures for passive liquid transportation on its lateral surfaces.

## Electronic supplementary material

Supplement 1 Video showing a z-stack of confocal microscope images of the non-structured MN after the wetting test. First, one sees the structures in the guiding channel. Later on, one sees the non-structured MN’s lateral faces and corners from the bottom (z = 0 μm) till z = 60 μm. (MP4 1447 kb)

Supplement 2 Video showing a z-stack of confocal microscope images of the structured MN after the wetting test. First, one sees the structures in the channels. Later on, one sees the structured MN’s lateral faces and corners from the bottom (z = 0 μm) till z = 90 μm. Note that the height range here is considerably larger than in Supplement 1. (MP4 1313 kb)

Supplement 3 A T-shaped guiding channel containing two different structured MNs (structures pointing downwards MN on the left, and structures pointing upwards on the right hand side). (**a**) The CAD design of the test sample. (**b**) Optical microscope image of the T-shaped channel and the two MNs before the wetting test. (**c**) Fluorescent microscope image taken in the red channel after the wetting test performed with a fluorophore-soap-water solution. Scanning electron microscope (SEM) images of the (**d**) MN with surface structures pointing downwards, and of the (**e**) MN with the surface structures pointing upwards, taken at a 45° view angle (scale bar corresponds to 20 μm). (**f**) Intensity profile along the white line in Supplement 3E (grey plot - before the wetting test, red plot - after the wetting test). For this certain wetting test a fluorophore concentration of 1 to 100 was used, therefore the signal intensity in image (**c**) is lower than in the Fig. 4 (PNG 5216 kb)

High Resolution Image (TIF 32858 kb)
