# Supplementary figures and images for: Bio-inspired microneedle design for efficient drug/vaccine coating
Source: Biomed Microdevices. 2019 Dec 16;22(1):8. doi: 10.1007/s10544-019-0456-z (PMC6915113; doi:10.1007/s10544-019-0456-z)

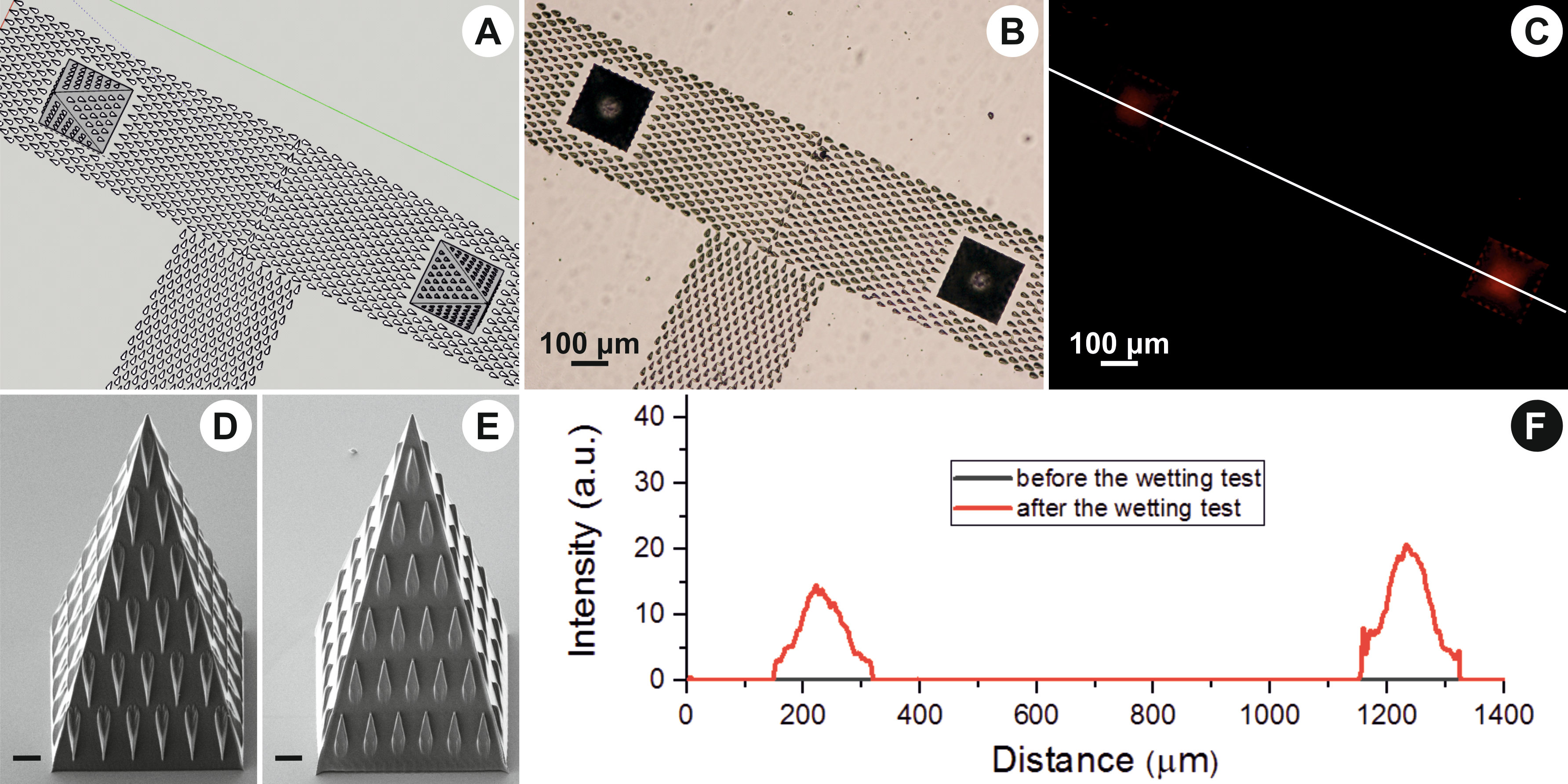

Supplement: Supplementary file 3 — A T-shaped guiding channel containing two different structured MNs (structures pointing downwards MN on the left, and structures pointing upwards on the right hand side). (a) The CAD design of the test sample. (b) Optical microscope image of the T-shaped channel and the two MNs before the wetting test. (c) Fluorescent microscope image taken in the red channel after the wetting test performed with a fluorophore-soap-water solution. Scanning electron microscope (SEM) images of the (d) MN with surface structures pointing downwards, and of the (e) MN with the surface structures pointing upwards, taken at a 45° view angle (scale bar corresponds to 20 μm). (f) Intensity profile along the white line in Supplement 3E (grey plot - before the wetting test, red plot - after the wetting test). For this certain wetting test a fluorophore concentration of 1 to 100 was used, therefore the signal intensity in image (c) is lower than in the Fig. 4 (PNG 5216 kb) [file 10544_2019_456_Fig7_ESM.png]
